# An Ishmaelite

**Published:** 1888-09-15

**Authors:** Leslie Keith

**Affiliations:** Author of "The Chilcotes," "Uncle Bob's Niece," "East and West," etc.


### An Ishmaelite.

By Leslie Keith,

Author of "The Chilcotes," "Uncle Bob's Niece," "East and West," etc.

(Continued from p. 378.)

### Chapter XXIV.—Fred's Triumph.

Jacob, in that beautiful story of a primitive courtship, thought seven years as nothing for the love he bore Rachel; but Arthur Eastgate found six months a severe trial to his patience.

In all that time he heard scarcely anything of his little love, whose hiding-place was kept jealously from him. If Proudie had been a duke and Arthur an unwelcome suitor, there could not well have been more firmness displayed on his side.

Arthur considered himself very ill used, and was at times inclined to resent this autocratic conduct on the part of a man who was so much his social inferior. "The fellow ought to have been glad enough of such a son-in-law," he thought at times, not unwilling to look upon himself as a very disinterested lover. But this mood only came uppermost when Miss Betsy had been particularly aggravating.

The family pride had its chief stronghold in this stern spinster's bosom; she would not suffer it to be supposed that Delia spent so much as a sigh upon the chances she had foregone. She took trouble to announce aloud that Delia was very happy and brisk, and enjoying her holiday life, thereby causing pangs of rage in the breasts of both her lovers.

A fellow-sympathy ought to have drawn Arthur to the cripple, to whom the doors of paradise were also closed; but, if the truth must be told, his feelings towards Tucker were more nearly allied to hate. Who was he that he dared aspire to Delia? He was not worthy to black her shoes. Arthur's behaviour at this time was far from heroic. Instead of forgetting Delia, as any well-regulated young fellow would have done, as Proudie made sure he would do, and thanking the kind fate that had interposed to save him from an unequal marriage, he but thought of her the more. Absence certainly strengthened the affections in his case. He hung about the shop and tried Barbara's serenity with his discontent and his melancholy airs, and he came very near to quarrelling with Fred over their difference on the same subject.

Arthur, who could be so wise for other people, was as other men are when they are the victims of love, and not a bit wiser, more reasonable, or more open to conviction.

The months which were disciplining Arthur were sifting and trying Fred too. There was no longer any question of his going away. There had never been a word between him and his employer on the matter, but after that revelation of his past which the revengeful little cripple brought about, Miss Betsy had given him to understand that he was expected to remain.

Fred thanked her humbly enough for the grace that allowed him this shelter; he caught at his reprieve as a condemned prisoner whose sentence is condoned. To be allowed to remain under the same roof, to wait on Barbara as a servant, was all he asked.

Miss Betsy still kept watch upon the pair, and sat—a stern figure of propriety—amidst the books; but all the Miss Betsys in the world could not prevent that which had happened. From the hour when Barbara bent to him in his humiliation, and gave him her hand in sign of fellowship and forgiveness, all his heart was hers. He would have died to serve her, but he never entertained any hope. To have spoken of his love would have seemed to him to dishonour her. There ought to be no stain upon the man who shared his life with her. Only the best was worthy of her.

And Barbara? She went her way and did her work with all the old cheerfulness and serenity; if there were times when she entertained sad visions, she kept them for the darkness and silence of her own chamber. Who can tell what battles she fought there; what prayers were wrung from her, what hopes and fears were conquered? Her calm face bore no record of those night watches, and the morning found her brave once more to face her daily task.

But there was a subtle, inevitable change in her relations with Fred, since the history of his past had been laid bare; in a thousand little ways, such as only a good woman knows the secret of, she helped and encouraged him. She cheered him by laughing with him over the humorous aspect of their task, the drolleries that crept up in the course of it, the queer idiosyncrasies of their clients, she stood by him at all times with an unshaken loyalty in her friendship, patient with him in his dark and fitful moods, ever ready to sustain and second him in his gropings after a better way of life. Was it any wonder he loved her and bitterly mourned the unworthiness that must seal his lips, and that placed her forever beyond his reach?

So those two love problems were working themselves out while the dark days sped by, and spring came back to make the world fair.

It is the season of joy when one is young, and Arthur, who, like other exiles, had fed himself on hope, took new courage with the advancing days.

One night he went home from the little shop almost exultant. Proudie had shown signs of a wavering will—of a relenting towards this persistent and importunate wooer.

"In another month you will have to give in," Arthur said; "even you can scarcely pretend to doubt my sincerity now."

Proudie rubbed the bald spot on his head, and his apple-like face puckered itself into lines of perplexity.

"You've not changed, Mr. Arthur, that I'll say for you; but then, may be, neither have I."

"Then it's quite time for you to begin," said Arthur with a laugh; "you've only a month left, and that's scant enough time for a dour Scotchman to do his repenting in."

He went his way with a heart that sung; the night was serene, and mildly starlit, and he felt, as egotistic lovers and poets always feel, as if old mother Nature were benignantly responding to his mood and rejoicing with him. He took no thought of the sad and embittered people to whom her jocund airs might appear a mockery. The moon and the stars, as all the world knows, are made for happy lovers; if only he could walk under their radiance with Delia at his side!

He went home taking his new happiness with him, and slept and dreamed of Delia, no doubt, and no warning vision came to tell him of the calamity that was about to descend upon the home he had left.

While he was wrapped in sleep and happy dreams, death had come very near to the old house in Gillespie Street. Fred, at no time an early sleeper, had been late in going to his room, and he had scarcely lost consciousness before a taint of smoke and charred wood scenting the air woke him. He got up and flung some clothes on hastily. His room was at the top of the tall, old house, the adjoining garret being occupied by Tucker. The other members of the family slept below.

The air in this upper region was clear enough as yet, but the smoke was curling up more thickly every moment. As he glanced down into the black gulf he was horrified to see a licking dart of flame, and then another, illuminating the darkness. The house was old, the woodwork like tinder, he knew what to expect. Almost before he could form the intention in his mind he had rushed downstairs and alarmed the sleepers there.

He went to the lower floors first, where Proudie and his wife and sister slept. There was still time for them to escape without danger, though the fire, which had its origin in the neighbouring shop, was steadily gaining a firm hold here too. Then he went back to the room which Barbara occupied. It was beneath his own. The smoke had come up more densely now, and the air was every moment gathering thickness, but there was a light burning in the room.

She had risen bewildered and oppressed, but with no distinct conception of her position, and had thrown a wrap about her. Her feet were bare, and her long hair hung loose over her shoulders; her face was pale, and her eyes had a startled, frightened look of appeal in them when they met his.

"What is it, Fred?" she said, unconsciously calling him by that name.

"You must come with me," he said. "Come quickly, there is time."

"Is there danger? —Father and mother——."

"They are safe—come."

As they stood looking at each other for that one brief instant, when peril seemed close upon their heels, and death, for all they knew, at the threshhold, they forgot the barrier that divided them. The vesture of convention fell away, and each saw the revealed secret in the other's face. They bent towards each other and their lips met tremblingly.

"Wrap this cloak about your mouth," he said, "and shut your eyes."

She obeyed without a word, and he lifted her in his strong arms; the flame had caught the stair here and there, and the fierce heat and smoke might have driven him back but for the burden he carried. He would have risked a thousand deaths for her. All his pent-up forces rushed out to meet the demand of the moment; he bore her safely through it all. The street was aroused, and the frightened, excited inhabitants had thronged out. Kind hands in plenty were ready to help the sufferers. Someone took Barbara from his arms and carried her away to her distracted parents.

The firemen had been summoned, but they had not yet come. Then a cry arose among the crowd that there was still one person in the burning house. Fred heard it, and he remembered Tucker. Before the people could remonstrate, or hold him from the mad act, he was back in the blazing house. How he reached the upper floor once more he never knew, but he got there. It was Fred's atoning hour. Who that knew or divined his story would have grudged him it?

The cripple flung himself upon him half mad with terror.

"Save me!" he screamed. "They have forgotten me. They have left me here to die!" He clung to Eastgate, he was frantic with the terrors of death that shook his soul. Fred, whom he had despised, maligned, reviled, had remembered him, but Tucker did not think of that. In his awful sense of desolation, he only knew that here was human fellowship, here was possible rescue. In the mortal hour of our need, are not all misunderstandings, offences, hatreds, swept away?

"I have come to help you if I can," said Fred wondering, not without a touch of scorn, at this abject surrender of all control. He himself was perfectly calm, he was almost happy.

He wrenched himself from the cripple's vice-like clutch to reach the door and there to calculate the chances of escape, but already he knew it was too late, the stair was crumbling under the devouring fire, a fiercer onslaught of flame and smoke drove him back to the clearer air by the open window. The room itself had not yet caught, there was a faint hope that one life, at least, might be saved if help from the outside but came soon.

He supported Tucker who had fallen half suffocated, half dead with fear to the floor, he lifted and helped him with a strong arm close to the window. The awful ruddy light illuminated the street and lit the pale upturned faces of the crowd below. Fred maintained his consciousness, his brain was active and clear as it had rarely been, and vivid in its workings. He saw his past unrolled before him in that brief five minutes that looked like so many years, while the crowd shouted with hoarse encouragement that the firemen were coming, he traced the beginnings of wilful sin that had wrought sorrow in the lives that were knit up with his own, and had made him an outlawed soul, with no path before him but the hard, stony way of penitence and renunciation, and now death was coming to end the struggle that that one moment of tremulous joy might have made too difficult. He faced it calmly, he cast himself humbly on the great compassion that never fails; the years of his humiliation had not been without their lessons, lessons which those who have not fallen in the fight may never learn.

The heat was every instant growing more stifling, he panted for breath; the crackle of the flame was in his ears, the leaping light blinded him. A terrible weariness and faintness took hold of him, an overpowering desire to lie down and be still.

He battled with it with a fierce clutch upon his ebbing energies, and kept his grasp of his unconscious companion. He held him there while the roar of the crowd came up to him. "The firemen are coming—they have come!"

Could he endure for another moment while they played upon the burning pile to make a way for the escape? He braced himself to a last supreme effort. He defied death. He endured till his companion was safe and then he knew no more.

*(To be continued.)*

